# Examining the Reasons for Patient No‐Show in an Outpatient Adult Rehabilitation Department in King Abdulaziz Medical City, Riyadh: A Cross‐Sectional Study

**DOI:** 10.1002/hsr2.70783

**Published:** 2025-04-29

**Authors:** Reouf B. AlOsaimi, Najla M. Aljehani, Turki Aljuhani

**Affiliations:** ^1^ Rehabilitation Services, King Abdulaziz Medical City Ministry of National Guard Health Affairs Riyadh Saudi Arabia; ^2^ Department of Public Health, College of Health Sciences The Saudi Electronic University Riyadh Saudi Arabia; ^3^ Department of Occupational Therapy, College of Applied Medical Sciences King Saud Bin Abdulaziz University for Health Sciences Riyadh Saudi Arabia; ^4^ King Abdullah International Medical Research Center Riyadh Saudi Arabia

**Keywords:** adults & rehabilitation, appointment, no‐show, outpatient

## Abstract

**Background:**

Patient no‐show is a long‐standing health issue that impacts the quality of healthcare services and resource utilization. No‐show creates a financial burden on healthcare systems and adds to the patient waiting list.

**Aims:**

The aim of the study is to investigate the prevalence of no‐show and the most common underlying reasons among adult patients at the physiotherapy and occupational departments.

**Methods:**

This cross‐sectional study was conducted at the Adult Rehabilitation Department of King Abdulaziz Medical City in Riyadh. Patients who failed to show up for their appointments were contacted via telephone. Data were collected using a telephonic questionnaire. Patient medical files were used to obtain demographic information and diagnoses.

**Results:**

During the study period, 336 patients did not show up to their appointments, representing 21% of the total appointments. Among the 336 patients, 140 were included in the study. The most common reason for no‐show was being sick on the day of the appointment (24%). Other common reasons were living outside Riyadh or needing an ambulance for transportation (17%). The third most common reason was an inconvenient appointment time or date (15%).

**Conclusion:**

The study findings suggest that to overcome this problem, patient awareness must be increased regarding the fact that their absence from therapy sessions can negatively affect their health states, prolong their illness, and worsen their condition.

## 1 Background

Missed or “No‐show” appointment poses a challenge for the hospital operation and administration side. Thus, it can lead to significant problems in hospital management, which impacts the healthcare system of the hospital. No‐show refers to a patient missing their scheduled appointment without notifying the clinic or hospital that they will not come [1]. When the challenge of no‐show is not solved and increases, this will lead to longer waiting times, higher healthcare expenditures, and lower healthcare system efficacy and efficiency [2, 3].

Globally, no‐show prevalence ranged from 12% to 80% in different healthcare settings [4]. In Saudi Arabia, studies reported the prevalence of no‐show between 11% and 30% depending on the location and services provided [5]. Multiple studies have identified various factors influencing no‐show rates, such as gender, age, service quality, number of preceding appointments, appointment lead times, and waiting times [6, 7, 8]. A second study showed that most no‐shows are more common among men, younger, and patients of lower socioeconomic status [9, 10]. An another study in Oman where the culture and lifestyle is similar to Saudi Arabia the following characteristics are frequently linked to no‐show events: younger adult, previous history of no‐shows, lower socioeconomic status, underinsured, being from a racial minority group, not married, place of residence distant from the clinic, out of work, problems with transportation, changed/improved health, and inability to leave school or work [11]. Similar factors are identified as predictors for no‐show in Saudi Arabia including, the young age group, appointment time and day, and transportations [12].

Thirty to forty percent of the patients seem to have chosen not to attend the scheduled appointment for personal reasons. The remaining 60%−70% of the patients were absent for various reasons that were beyond their control, like transportation issues or forgetting the appointment [11].

No‐show scheduled appointments are a missed healthcare designated time slots and resources which can negatively impact the utilization of space and human recourses. More importantly they can delay diagnosis or treatment which can impact the patient's health condition [4].

Furthermore, generated revenue also decreases due to the reduction in the system's operational efficiency. For example, a study estimated that 67,000 no‐show appointments cost the healthcare system approximately $7 million [7]. Thus, lowering the prevalence of patient no‐show would not only enhance hospitals' efficiency but also increase revenues [13].

While no‐show prevalence various based on the clinical settings, rehabilitation services could have a very high prevalence due to the multiple sessions and appointments required. Yet there are very few studies that examine the prevalence rate and reasons within the rehabilitation services. In a large study that included 828 physical therapy commercial centers the percentage of missing at least one appointment was 73% [14]. While another study found that the rate of no‐show was 20.6% in a public health provider center [15]. Multiple factors cause the no‐show in rehabilitation in private (commercial center), the most common reason was insurance coverage other reasons were time between visits and scheduling date, forgetters, and transportation.

In Saudi Arabia, research examining the no‐show prevalence is scarce, along with research on the underlying reasons for this phenomenon in adult outpatient settings in general and in rehabilitation services specifically. Identifying the reasons behind patients missing appointments could provide guidance on how to reduce no‐show prevalence, thus improving overall patient care. Therefore, the aim of this study was to identify the prevalence of no‐show and the most common reasons for it among adult patients at the outpatient rehabilitation department at King Abdulaziz Medical City.

### 1.1 Research Objectives

The following steps were taken during the study to answer the research questions:

1. Identify the prevalence of no‐show among adult patients at the outpatient rehabilitation department at King Abdulaziz Medical City.
2. Identify the common reasons for no‐show among adult patients at the outpatient rehabilitation department at King Abdulaziz Medical City.

## 2 Materials and Methods

### 2.1 Study Design

A cross‐sectional study was conducted on adult patients who received physical therapy and occupational therapy treatment at King Abdulaziz Medical City outpatient clinics in Riyadh.

### 2.2 Setting

This study was conducted on both male and female patients who attended adult clinics to receive physical therapy and occupational therapy treatment sessions at King Abdulaziz Medical City outpatient clinics in Riyadh. King Abdulaziz Medical City in Riyadh (KAMC‐RD) opened its doors in 1982, and it is considered one of the most comprehensive medical facilities in Saudi Arabia. The study was carried out in adult outpatient rehabilitation clinics. These clinics has more than 15 therapists between physical and occupational therapists who collaborate to provide integrated, patient‐centered care. The clinics run from Sunday to Thursday from 8:00 a.m. to 5:00 p.m., with a minimum of seven patients booked per day for each therapist schedule, with an approximate total of 105 patients per day.

### 2.3 Sample Technique

A consecutive sampling method was used in this study. Adult participants who received rehabilitation services and did not show up for their appointment during the month of May 2023 were contacted through phone calls. The month of May was selected randomly and as soon as the study was approved, no public holidays were presented in the selected month as well. Demographic data (age and gender) were collected using documents and records concerning the no‐show prevalence in adult outpatient rehabilitation clinics and were collated in an Excel sheet.

### 2.4 Inclusion and Exclusion Criteria

- All booked male and female outpatients who attended adult clinics and received physical therapy and occupational therapy services from all clinics related to the rehabilitation department, with a no‐show record during the data collection period.
- To be included, the clients have to attend both or at least one of the rehabilitation services.

Exclusion criteria:

- Patients who are younger than 14 years old.
- Inpatients.
- Any patient without a no‐show record during the period of data collection.

### 2.5 Data Collection Tools

The data collection tool used in this study was a telephone questionnaire. The questionnaire was adopted and modified from a previous study done by Alawadhi et al. [5, 16]. The questionnaire consists of five questions. The first question is related to the reason for absence from medical appointments in 15 categories. The second question concerns transportation (either by themselves, with a husband, or with a relative). According to the General Authority for Statistics, the number of Riyadh residents reached 8.4 million in 2018; consequently, the number of automobiles has significantly increased [17]. The Ar‐Riyadh Development Authority (ADA) estimated that private automobiles are used for more than 92% of daily travel. Most residents of Riyadh have never used public transportation and are used to using their own cars for everyday commuting [18]. Therefore, it was essential to include transportation in the study questionnaire to determine whether participants have a license and/or have a car. Additionally, as females were part of the study, and since they were only allowed to drive beginning in 2018, it was important to identify how they attended their appointments, whether they were accompanied by a relative or drove by themselves [17]. The third question concerns the effective utilization of the hospital reminder system (patients receiving SMS reminder messages on their mobile phones about the appointment and trying to call the hospital to cancel the appointment and rebook). The fourth is about job status (employed, not employed, student). The final question is an open question to receive suggestions for improving the appointment system. A pilot test of the survey was conducted on a subset of our intended population. The pilot test was then revised based on the information gained from it. Finally, the questionnaire was used in the study (Appendix A). Demographic variables, including age and gender, were retrieved from the patient's electronic medical records. The patients with no‐shows were interviewed through a telephone call from the rehabilitation department at the end of the same day, with consent obtained orally before the interview. In case of no answer, the same patients were contacted in succeeding days until the end of the data collection period. These interviews were witnessed by an external member to ensure the integrity of the data. The external members were part of the hospital facility and mostly were the main treating therapists of the patient or unit supervisors. Their role was to introduce the participants to the research team, all external members were qualified treating therapists or clinical supervisors. Each phone call took around 5 min. Patients were asked about the reason for no‐show to their appointments.

### 2.6 Data Analysis

The data were collected and entered into Microsoft Excel before being exported into the Statistical Package for the Social Sciences (SPSS) Version 28.0.1.0 for statistical analysis. Tables and figures were used to present the results. The results were defined using descriptive and analytical statistics.

### 2.7 Ethical Considerations

The researcher obtained ethical approval from the King Abdullah International Medical Research Center (SP23R/022/04). The participants were informed about the purpose of the study in detail. Researchers ensured that the participants whose data were being used in the study provided their consent for their data to be utilized for research purposes. The participants were informed that they were allowed to withdraw from the study if they did not want to participate. The researchers took steps to protect the confidentiality and privacy of the participants whose data were being used in the study. This included anonymizing the data and obtaining permission to use the data from the participants.

## 3 Results

The results of the study had five aspects: participant demographics, prevalence of no‐shows, reasons for missing medical appointments, ways of transportation, and the effective utilization of the hospital communication system. Each of these categories is explained below based on the study questionnaire.

### 3.1 Participant Demographics

A total of 140 patients participated in the study. Among them, 59% were female, while 41% were male. Most of the participants were in the age category of 41–60 years old, which represents around 33.6% of all ages. In total, 78 participants (56%) were unemployed, and 45 participants (32%) were employed. There were 17 students (*n* = 17), representing around 12% of the study participants. Table 1 shows the demographic details of the study.

**Table 1:** Participant demographics.

	Number	Percentage
Gender		
Male	57	41%
Female	83	59%
Total	140	100%
Occupation		
Employee	45	32%
Unemployed	78	56%
Student	17	12%
Total	140	100%
Age group		
14−20	14	10%
21−40	46	33%
60−41	47	34%
61 and above	33	24%
Total	140	100%

### 3.2 Prevalence of No‐Shows

The total number of appointments for physical and occupational therapy was 1712. Through follow‐ups and monitoring of these appointments, it was found that 366 patients missed their appointments, representing approximately 21% of the appointments. From this no‐show cohort, 140 patients were included in the study as they responded to the telephone call. Table 2 presents the prevalence of no‐shows among the study participants.

**Table 2:** The prevalence of no‐shows during the month of May among adult patients at the outpatient rehabilitation department at KAMC.

No of appointments during the month of May 2023	No of no‐show during the month of May 2023	No of population during the month of May 2023
1712	366	140
(100%)	(21%)	(39%)

### 3.3 Reasons for No‐Show

After exporting and plotting the data of the reasons for no‐show into SPSS, it is clear from Table 3 that the reasons for patient no‐show varied. The most frequently reported reason was “I was sick on the day of the appointment,” with 25% of the study participants citing this reason. Transportation struggles ranked as the second most reported reason, with 16% of the participants mentioning that they had difficulties getting transportation to the hospital. The least frequent reason was related to the absence of symptoms on the day of the appointment, with 1.4% of the participants choosing the category of “I was feeling okay on the day of the appointment” as the reason for no‐show. Additionally, 17% mentioned other reasons that were not included in the questionnaire, the most important of which was having COVID‐19 and needing an ambulance to transfer them to hospital appointments.

**Table 3:** Reasons for no‐show among study participants.

1.	I forgot about the appointment.	5	4%
2.	The appointment was set at an inconvenient time/date.	21	15%
3.	I had family commitments.	6	4%
4.	I was sick on the day of the appointment.	34	24%
5.	I was feeling okay on the day of the appointment (absence of symptoms).	2	1.4%
6.	I had difficulties getting transportation to the hospital.	23	16%
7.	I overslept.	0	0%
8.	I did cancel the appointment before the date of the appointment.	0	0%
9.	I rescheduled the appointment for another day.	4	3%
10.	I could not get time off work.	17	12%
11.	I had another appointment at a different hospital.	3	2%
12.	I waited too long for the appointment.	0	0%
13.	Not having a companion.	1	0.7%
14.	No reason given.	0	0%
15.	Other	24	17%
	Total	140	100%

### 3.4 Transportation

Table 4 shows that around 42% of the study participants had a driving license, while 45% of the participants had a car and more than half 58% of the female participants' do not have a driver license. Table 5 illustrates the way female patients attended their appointments; the results show that the majority 60% relay on relative to drive them for their appointments.

**Table 4:** Prevalence of having a license/car.

Sentence	Yes		No	
Do you have a driver's license?	58	(41.5%)	82	(58.5%)
Do you have a car?	63	(45%)	77	(55%)

**Table 5:** The way female patients attend their appointments.

By myself	With relative	With husband	Taxi	Total
12	50	8	13	83
(14%)	(60%)	(10%)	(16%)	(100%)

### 3.5 Hospital Communication

Table 6 presents the data on the effective utilization of communication with respect to whether participants did receive reminders regarding their appointments and whether they tried to use the available communication methods to cancel or rebook their appointments. In the current study, 91% of the participants received appointment reminder messages. The majority of the sample (68%) did not try to call the hospital to cancel their appointments beforehand, and only 32% called the department. A similar trend was found concerning the rescheduling of appointments: 77% of the participants did not try to rebook for a new appointment.

**Table 6:** Effective utilization of communication.

Question	Yes		No	
Did you receive an SMS reminder message on your mobile phone about your appointment?	128	(91%)	12	(9%)
Did you try to call the hospital to cancel your appointment?	45	(32%)	95	(68%)
Did you rebook for a new appointment?	32	(23%)	108	(77%)

### 3.6 The Collaboration Between Gender and the Reasons of No Show

To perform a chi‐square test for independence between the reasons for no‐shows and patient gender, the raw data summarized was used. The null hypothesis is that there is no association between the reasons for no‐shows and patient demographics, while the alternative hypothesis is that there is an association.

To perform the chi‐square test for independence, a contingency table that summarizes the counts of reasons for no‐shows by gender and age group was created and shown in Table 7.

**Table 7:** Reasons of no show per gender.

Reason for no‐show\gender	Female	Male	Total
I was sick on the day of the appointment.	23	11	34
Other	15	9	24
I had difficulties getting transportation to the hospital.	16	7	23
The appointment was set at an inconvenient time/date.	16	5	21
I could not get time off work.	3	14	17
I had family commitments.	2	4	6
I forgot about the appointment.	4	1	5
I rescheduled the appointment for another day.	3	1	4
I had another appointment at a different hospital.	1	2	3
I was feeling okay on the day of the appointment.	0	2	2
Not having a companion.	0	1	1
Grand total	83	57	140

The expected frequencies were calculated, and then the chi‐square test was conducted (Table 8). As a start, a calculation for the expected frequencies was done:

1. Calculate row and column totals.

- Row totals: Female (83), male (57)
- Column totals:
  ○ I was sick on the day of the appointment (34)
  ○ Other (24)
  ○ I had difficulties getting transportation to the hospital (23)
  ○ The appointment was set at an inconvenient time/date (21)
  ○ I could not get time off work (17)
  ○ I had family commitments (6)
  ○ I forgot about the appointment (5)
  ○ I rescheduled the appointment for another day (4)
  ○ I had another appointment at a different hospital (3)
  ○ I was feeling okay on the day of the appointment (2)
  ○ Not having a companion (1)

2. Calculate the expected frequency for each cell using the formula:

$$\mathrm{Expected}\;\mathrm{Frequency}=\frac{\mathrm{Row}\;\mathrm{Total}\times \;\mathrm{Column}\;\mathrm{Total}}{\mathrm{Grand}\;\mathrm{Total}}$$

**Table 8:** Expected frequencies for reasons of no show per gender.

Reason for no‐show\gender	Female	Male	Grand total
I was sick on the day of the appointment.	15.43	18.57	34
Other	10.86	13.14	24
I had difficulties getting transportation to the hospital.	12.27	10.73	23
The appointment was set at an inconvenient time/date.	10.71	10.29	21
I could not get time off work.	8.14	8.86	17
I had family commitments.	2.86	3.14	6
I forgot about the appointment.	2.29	2.71	5
I rescheduled the appointment for another day.	1.71	2.29	4
I had another appointment at a different hospital.	1.29	1.71	3
I was feeling okay on the day of the appointment.	0.86	1.14	2
Not having a companion.	0.43	0.57	1
Grand total	83	57	140

To perform the chi‐square test using the expected frequencies, the observed and expected frequencies for each cell were compared. The chi‐square statistic helped determine whether there is a significant association between reasons for no‐shows and gender. A calculation of the chi‐square value was completed, and the test was performed presented in Table 9.

**Table 9:** Observed frequencies & expected frequencies for reasons of no show per gender.

Reason for no‐show\gender	Female (observed)	Male (observed)	Female (expected)	Male (expected)
I was sick on the day of the appointment.	23	11	15.43	18.57
Other	15	9	10.86	13.14
I had difficulties getting transportation to the hospital.	16	7	12.27	10.73
The appointment was set at an inconvenient time/date.	16	5	10.71	10.29
I could not get time off work.	3	14	8.14	8.86
I had family commitments.	2	4	2.86	3.14
I forgot about the appointment.	4	1	2.29	2.71
I rescheduled the appointment for another day.	3	1	1.71	2.29
I had another appointment at a different hospital.	1	2	1.29	1.71
I was feeling okay on the day of the appointment.		2	0.86	1.14
Not having a companion.		1	0.43	0.57
Grand total	83	57	83	57

To calculate the chi‐square statistic, we use the formula:

$$X^{2}=\sum \left(\frac{{\left(\text{Observed}-\text{Expected}\right)}^{2}}{\text{Expected}}\right)$$

$$=X^{2}=\left(\frac{{\left(23-15.43\right)}^{2}}{15.43}\right)+\left(\frac{{\left(15-10.86\right)}^{2}}{10.86}\right)+\left(\frac{{\left(16-12.27\right)}^{2}}{12.27}\right)+\left(\frac{{\left(16-10.71\right)}^{2}}{10.71}\right)\ldots +\left(\frac{{\left(0-0.43\right)}^{2}}{0.43}\right)$$

= 4.03

The degrees of freedom (df) for a chi‐square test in this case is calculated as (Number of rows−1) × (number of columns−1) (number of rows−1) × (number of columns−1), which is (11 − 1) × (2 − 1) = 10 (11 − 1) × (2 − 1) = 10.

Finally, the calculated chi‐square value of 4.03 to the critical chi‐square value at a chosen significance level (e.g., 0.05) with 10 df were compared to determine whether the association between reasons for no‐shows and gender is statistically significant.

The calculated chi‐square statistic is approximately 4.03, with 10 df. To determine the statistical significance of this result, a comparison to the critical chi‐square value at a chosen significance level (e.g., 0.05) with 10 df was done.

Using a chi‐square distribution table, the critical chi‐square value at a 0.05 significance level and 10 df is approximately 18.31. Since 4.03 is less than 18.31, the null hypothesis was not rejected. This means that there is no significant association between reasons for no‐shows and gender based on the chi‐square test.

In summary, the chi‐square test does not provide evidence to suggest that reasons for no‐shows are significantly associated with patients' gender in this data set.

### 3.7 The Collaboration Between Age Group and the Reasons of No Show

To perform a chi‐square test between reasons for no‐shows and age groups, a calculation of the expected frequencies for each cell in the contingency based on the assumption of independence between reasons for no‐shows and age groups was done. After that, a calculation of the chi‐square statistic was done to determine the *p*‐value to assess the significance of the association (Table 10).

**Table 10:** Reasons of no show per age group.

Reason for no‐show\gender	14−20	21−40	41−60	61 and older	Total
I was sick on the day of the appointment.	0	18	7	9	34
Other	0	0	6	18	24
I had difficulties getting transportation to the hospital.	1	0	22	0	23
The appointment was set at an inconvenient time/date.	9	10	0	2	21
I could not get time off work.	0	11	6	0	17
I had family commitments.	0	6	0	0	6
I forgot about the appointment.	4	1	0	0	5
I rescheduled the appointment for another day.	0	0	4	0	4
I had another appointment at a different hospital.	0	0	0	3	3
I was feeling okay on the day of the appointment.	0	0	2	0	2
Not having a companion.	0	0	0	1	1
Grand total	14	46	47	33	140

To calculate the expected frequencies for each cell in the Table 11, the following steps were taken:

**Table 11:** Expected frequencies for reasons of no show per age group.

Reason for no‐show\gender	14−20	21−40	41−60	61 and older	Total
I was sick on the day of the appointment.	1.71	5.63	5.76	4.90	18
Other	1.29	4.24	4.35	3.12	12
I had difficulties getting transportation to the hospital.	1.46	4.81	4.93	4.19	16
The appointment was set at an inconvenient time/date.	1.08	3.56	3.65	2.71	11
I could not get time off work.	0.92	3.02	3.09	2.30	9
I had family commitments.	0.29	0.95	0.97	0.72	3
I forgot about the appointment.	0.92	3.02	3.09	2.30	9
I rescheduled the appointment for another day.	0.54	1.77	1.81	1.35	5
I had another appointment at a different hospital.	0.38	1.25	1.28	0.95	3
I was feeling okay on the day of the appointment.	0.23	0.76	0.78	0.58	2
Not having a companion.	0.08	0.26	0.27	0.20	1
Grand total	9	30	31	23	93

Calculate the row totals for each reason and the column totals for each age group.

Calculate the overall total.

Calculate the expected frequency for each cell using the formula:

$$\mathrm{Expected}\;\mathrm{Frequency}=\frac{\mathrm{Row}\;\mathrm{Total}\times \;\mathrm{Column}\;\mathrm{Total}}{\mathrm{Grand}\;\mathrm{Total}}$$

To perform the chi‐square test between reasons for no‐shows and age groups, the observed and expected frequencies from the contingency table were used in Table 12. The chi‐square statistic is calculated as:

$$X^{2}=\sum \left(\frac{{\left(\mathrm{Observed}-\mathrm{Expected}\right)}^{2}}{\mathrm{Expected}}\right)$$

**Table 12:** Chi‐square test results for Reasons of no show per gender.

Reason for no‐show\gender	14−20	21−40	41−60	61 and older	Total
I was sick on the day of the appointment.	0.45	2.18	0.49	1.14	4.26
Other	1.10	0.92	0.04	3.53	5.59
I had difficulties getting transportation to the hospital.	0.27	4.40	4.33	2.75	11.75
The appointment was set at an inconvenient time/date.	7.74	2.13	2.87	2.55	15.29
I could not get time off work.	8.74	0.12	1.51	6.69	17.06
I had family commitments.	0.04	2.17	0.95	2.52	5.68
I forgot about the appointment.	1.63	0.25	2.67	4.11	8.66
I rescheduled the appointment for another day.	4.58	0.97	0.07	2.58	8.20
I had another appointment at a different hospital.	0.46	0.30	0.30	0.66	1.72
I was feeling okay on the day of the appointment.	0.08	1.19	0.42	0.05	1.74
Not having a companion.	0.01	0.02	0.16	0.11	0.30
Grand total	24.10	14.85	13.80	29.68	82.42

To calculate the df and find the *p*‐value associated with this chi‐square statistic to assess the significance of the association between reasons for no‐shows and age groups.

To find the *p*‐value for the chi‐square test between reasons for no‐shows and age groups, there is a need to determine the df. The df for a chi‐square test in this context are calculated as:

Df = (r − 1) × (c − 1)

Where:

r = Number of rows (reasons) in the contingency table

c = Number of columns (age groups) in the contingency table

(11 – 1) x (4 − 1) = 30

With df = 30, the *p*‐value can be obtained from a chi‐square distribution table to find the exact *p*‐value associated with the calculated chi‐square statistic. The critical value of chi‐square with 30 df at a significance level of 0.05 (commonly used threshold) is approximately 50.892.

Since the calculated chi‐square statistic (82.42) is greater than the critical value (50.892), it can conclude that there is a significant association between reasons for no‐shows and age groups in the given data. The *p*‐value associated with this chi‐square statistic is extremely small, indicating strong evidence against the null hypothesis of independence between reasons for no‐shows and age groups.

## 4 Discussion

In the current study, the no‐show prevalence was around 22% of the total scheduled appointments. While nonattendance prevalence ranged from 12% up to 80%, in different international healthcare settings. Our result, is considered within the normal range [4]

In our study, the most reported reason for no‐show was being sick on the day of the appointment, with 25% of the participants mentioning that as their reason. This result is different from that of Alawadhi et al., who found that the most frequently reported reason for missed appointments was transportation difficulties, at around 24% [16]. On the other hand, our result coincided with that of Cohen‐Yatziv et al., who found that illness was among the leading reasons for ambulatory clinic no‐shows together with forgetfulness and administrative issues [19]. Being absent due to sickness is a critical issue in healthcare. Therefore, it is crucial for healthcare facilities to encourage their patients to contact the facility whenever they want to cancel their appointments, thus saving the slot for another patient. Indeed, patients need to be aware that absence from therapy sessions negatively affects their health states and prolongs their illness. In doing so, the number of no‐shows might be reduced.

“Other reasons” came in second, with a prevalence of 17.1%. One of the primary causes was not receiving the appointment message. This reason was also cited in the study of Al‐Awadhi et al., where “no SMS received” was listed as one of the most common excuses for missing an appointment [16]. According to Su et al., outpatient appointment systems are a key component of hospital resource interventions because they significantly influence patient experiences and the effective utilization of hospital services [1]. The reminder system in the rehabilitation department of National Guard Health Affairs sends a reminder message to the patient's cell phone before the appointment time. At the same time, the accuracy of the system might be in doubt, since some patients may change their phone number without notifying the hospital.

One of the interesting reasons was the patients' need for an ambulance to be transferred to the hospital. This was also reported by Marbouh et al., who found that one of the reasons for missed appointments among their participants was the need for an ambulance [4]. This finding highlighted the importance of interdisciplinary collaborations to improve appointments attendance and clients experience especially between the rehabilitation staff and emergency department.

Having difficulties with getting transportation to the hospital as a reason for no‐show was cited by 16.4% of participants. This result is in alignment with the results of Alkomos et al., who found that the reasons for not attending hospital appointments could be related to difficulty in obtaining transportation and distances between clinics and patients' homes [20]. Transportation was again identified as the most frequent reason in the study of Al‐Awadhi et al. at the Royal Hospital in the Sultanate of Oman. In their study, patients mentioned that they traveled long distances, since they lived far away from the hospital [16]. This could be a valid reason if the appointment frequency is high, particularly if the patient is not satisfied with the therapy or with the time spent in the sessions, which will make patients weigh the travel time versus the limited benefits of the treatment sessions.

Our results indicate that 42% of the study participants had a driving license. This may be because females represented around 59% of the study population when females were only recently allowed to drive in the Kingdom in 2018. This might also explain why 60% of female patients attended medical appointments with relatives. This difference in the percentage is similar to the findings of Al‐Awadhi et al., which showed that only 31% of the female participants owned a car, compared with 67% of their male counterparts. According to Al‐Awadhi et al., this is a frequent problem that might be related to the Middle East's cultural characteristics [16]. Therefore, special care should be taken when scheduling appointments for female patients, and patients should be encouraged to arrange transportation in advance or to postpone if attending the appointment is not possible.

The effective utilization of hospital communication was positively clear in the current study, with 91% of participants receiving appointment reminder messages. This indicates a strong and effective reminder system. However, 9% of the participants did not receive the message, which might have increased the possibility of them missing their appointments. Alawadhi et al. also reported that 75% of their participants received an SMS reminder for their appointment [20]. Thus, it was recommended in their study to improve the SMS reminder system by either using phone call reminders or increasing the SMS reminder frequency while providing patients with an accessible, easy‐to‐use appointment scheduling system. In addition, a more recent study found an increase in false no‐shows where the patients were reported as no‐show while in fact, they attended their appointment. Thus, happened as the Ministry of Health was transition from the backlog of Mawid two‐way user system of patients and healthcare staff to one‐way patient user scheduling app [21].

Another reason for not receiving the reminders is the fact that some patients use multiple contact numbers or fail to update their numbers, which causes registration issues. One example is when women typically give their male relative's (husband, brother, or father) phone number as their contact number. Typically, a chaperone joins female patients during their hospital appointments. Thus, the male chaperone will also provide his phone when asked by hospital employees at the registration desk to verify the appointment and update the contact information. As a result, any reminders going forward are not sent to the female patient directly but instead to the phone number provided at registration, which might increase the change of missing appointments [8, 16].

Regarding rescheduling, 77% of our sample failed to call the hospital before the time of the scheduled appointment to book a new appointment. Wasted time and resources in healthcare can have an adverse effect on how well equipment and staff are used and may also have an impact on patients' health because of a delayed diagnosis or course of treatment [4]. To address this, it is recommended that healthcare facilities focus on facilitating and explaining the methods of rescheduling appointments to patients, such as calling the department extension or through the development of a patient services application.

Another effective strategy that can reduce no‐show appointments is utilizing a multidisciplinary team approach, by scheduling appointments on the same day, if possible, for both occupational and physical therapy sessions. A multidisciplinary approach is known to improve patient outcomes and improve the patient's satisfaction level of services provided [22]. Thus, it can be utilized in reducing patient no‐shows. In addition, the utilization of telerehabilitation in specific conditions can help in reducing the burden on the patients. Telerehabilitation intervention was found to be similar to traditional rehabilitation interventions in patients with chronic respiratory diseases [23], stroke [24], and following orthopedic surgery [25]. Another study within the context of Saudi Arabia proves the usefulness of telerehabilitation in reducing pain in patients with musculoskeletal conditions [26].

Nonattendance to scheduled hospital outpatient appointments may compromise healthcare resource planning, which ultimately reduces the quality of healthcare provision by delaying assessments and increasing waiting lists [2]. The risk of nonattendance can be adequately estimated using patient information stored in medical records which allows prioritizing interventions, such as phone call reminders, to reduce nonattendance prevalence [2]. Moreover, patient needs to have the choice to select an appropriate appointment reminder and be aware of the different choices available [20]. Since some reasons for no‐shows are unavoidable, patients should be educated of the significance of calling to cancel an appointment.

With the advancement of technologies in the healthcare system and the raise of the artificial intelligent (AI) applications within the health system, tracking and identifying potential no‐show patients can be beneficial. A recent review paper showed that technologies that has been used in the healthcare to track no‐show patients in controlling some no‐show appointments, promote patient engagement, and increase health literacy [27]. Furthermore, the utilization of AI can help in predict no‐show patient thus identify and reduce the barriers leading to no‐show [5]. The application of AI in predicting no‐show patients has been conducted in Saudi Arabia with early positive results [28].

### 4.1 Strengths and Limitations

This research has its strengths and limitations. The main strength is that the study followed a quantitative design, which enabled the exploration of the reasons for no‐show. The detailed description of the patient's experiences gave an understanding of the reasons for no‐show. Through this study, hypotheses for future research can be generated. This is also the first research study done on missed appointments among adult patients attending rehabilitation clinics in King Abdulaziz Medical City in Riyadh. On the other hand, social desirability bias might be one limitation, since patients might feel uncomfortable sharing the exact reason for no‐show on the phone, or they might tend to answer what they believe the researcher would prefer them to say. In addition, the response rate of 38.25% (140 out of 366 patients) raises concerns about the representativeness of the sample; thus, our generalizability is a limitation. The current study was limited to one department in one hospital in the city of Riyadh. As a result, the findings from this study might be specific to the adult rehabilitation department in King Abdulaziz Medical City and not generalizable to other settings.

## 5 Conclusions

In conclusion, no‐shows affect the effectiveness of the medical services provided and scheduling efficiency. To guarantee that patients receive the greatest possible health benefits, interventions that increase patient's attendance to clinics need to be developed. Understanding patients' reasons for skipping appointments can also help build successful solutions. These missed appointments waste resources; thus, decreasing no‐show prevalence is a significant task for healthcare institutions. Improved awareness can decrease hospital no‐show prevalence. It also has the potential to have a positive impact on the provider's time, hospital revenue, patient outcomes, and the overall healthcare system.

## Author Contributions

**Reouf B. AlOsaimi:** methodology, investigation, formal analysis, writing – original draft, writing – review and editing, resources. **Najla M. Aljehani:** supervision, project administration, writing – original draft, writing – review and editing, methodology. **Turki Aljuhani:** writing – original draft, resources, writing – review and editing. All authors have read and approved the final version of the manuscript.

## Ethics Statement

This study was presented to the Research Council of King Abdullah International Medical Research Center and was approved before conducting the study with the code of ethics under the number SP23R/022/04 (Approval Date: 2023‐04‐13). All procedures performed in this study were performed in accordance with the ethical standards contained in the Declaration of Helsinki and its subsequent amendments or comparable ethical standards.

## Consent

Informed consent was obtained from all participants in the study.

## Conflicts of Interest

The authors declare no conflicts of interest.

## Transparency Statement

The lead author, Reouf B. AlOsaimi, affirms that this manuscript is an honest, accurate, and transparent account of the study being reported; that no important aspects of the study have been omitted; and that any discrepancies from the study as planned (and, if relevant, registered) have been explained.

## Data Availability

The data that support the findings of this study are available from the corresponding author upon reasonable request. Data sets are available through the corresponding author upon reasonable request. Reouf B. AlOsaimi, Rehabilitation Services [King Abdulaziz Medical City, Ministry of National Guard Health Affairs, Riyadh, Saudi Arabia. Contact details: Email: alosaimireouf@gmail.com. Phone: 00966548357928] had full access to all the data in this study and takes complete responsibility for the integrity of the data and the accuracy of the data analysis.

## Appendix A

Phone survey questionnaire form.

Reasons for no‐show in outpatient adult rehabilitation in King Abdulaziz Medical City, Riyadh: Perspectives of patients in a survey.

Please complete the case report form for each patient when conducting the survey.

Make sure that you obtain the patient's verbal informed consent at the beginning of the call.

**Table:**

Question 1. You have missed your scheduled appointment, as shown in the hospital system. Could you please identify/state the reason for not attending the appointment?

Patient Response:

Which category best describes the reason given by the patients from the following statements?

1. I forgot about the appointment.
2. The appointment was set at an inconvenient time/date.
3. I had family commitments.
4. I was sick on the day of the appointment.
5. I was feeling okay on the day of the appointment.
6. I had difficulties getting transportation to the hospital.
7. I overslept.
8. I did cancel the appointment before the date of the appointment.
9. I rescheduled the appointment for another day.
10. I could not get time off work.
11. I had another appointment in a different hospital.
12. I waited too long for the appointment.
13. Not having a companion.
14. No reason given.
15. Others.

**Table:**

Question 2.
Response number from the above list: (To be completed by a member of the research team)

A. Do you have a driver's license? Yes No
B. Do you have a car? Yes[] No[]
  If the patient is female and has a car, please ask the following question:
C. When you attend your appointment, with whom do you come to the appointment?
  By Myself[] With Relative[] With Husband[]
  If the patient has no car, please ask the following question.
D. How do you usually go to the appointment?

With Relative/Friend[] Taxi[]

**Table:**

Question 3. Question 2.

A. Did you receive an SMS reminder message on your mobile phone about your appointment?
B. Yes[] No[]
C. Did you try to call the hospital to cancel your appointment? Yes[] No[]
D. Did you rebook for a new appointment? Yes[] No[]

**Table:**

Question 4. Question 2.

A. Are you: Employed[] Not employed[] Student[]

**Table:**

Question 5. Question 2.

How can we improve the appointment system?

Thank you for your time in answering the questions. Your identity will be anonymous, and your participation in this survey will not affect the way you receive health services at KAMC.

Have a good day.
